# *CURLY LEAF* is required for the auxin-dependent regulation of 3-dimensional growth specification in *Physcomitrium patens*

**DOI:** 10.17912/micropub.biology.000797

**Published:** 2023-04-17

**Authors:** Rency J. Raquid, Richard Jaeger, Laura A. Moody

**Affiliations:** 1 Department of Biology, University of Oxford, South Parks Road, Oxford, OX1 3RB, United Kingdom

## Abstract

The *no gametophores 4* ( *nog4-R* ) mutant cannot make the transition from 2-dimensional (2D) to 3-dimensional (3D) growth in *Physcomitrium patens* and forms side branch initials that are largely fated to become sporophyte-like structures. We describe the three different developmental trajectories adopted by the *nog4-R* mutant, all of which result in indeterminate growth and defects in cell division plane orientation. A candidate gene approach confirmed that the causative mutation resided in the *CURLY LEAF* gene, and we highlight a previously uncharacterized role for *CURLY LEAF* in maintaining auxin homeostasis in *P. patens* .

**Figure 1. Developmental characterization of the no gametophores 4 -R ( nog4-R ) mutant.**
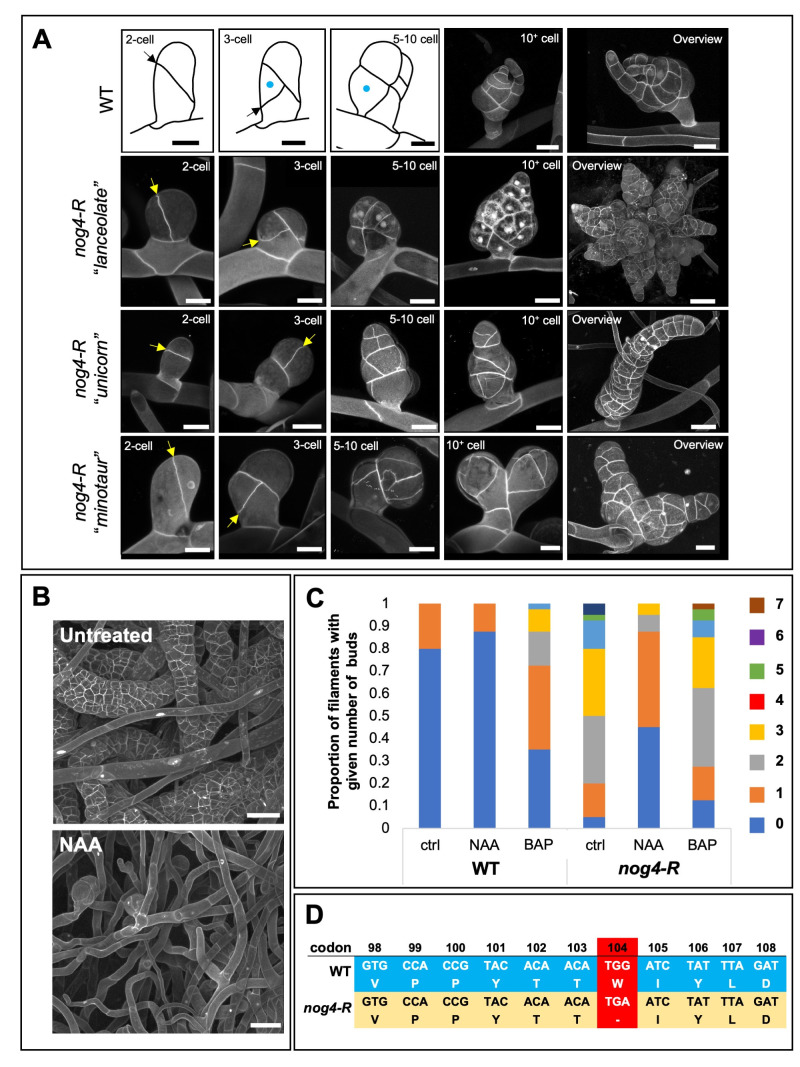
A) The three developmental trajectories observed in the *nog4-R* mutant ( *lanceolate* , *unicorn* and *minotaur* ) at the 2-cell, 3-cell, 5-10 cell and 10+ cell stages and an overview of an intermediate stage of development. A schematic has been included to enable comparisons more clearly with the predictable pattern of development observed in wild type. Developing ‘buds’ have been stained with propidium iodide and imaged using confocal microscopy. In the 2-cell and 3-cell images, the most recent divisions have been indicated by yellow arrows. A blue dot denotes the rhizoid initial cell. Scale, 20 µm. B) Representative images of one month old *nog4-R* tissues grown in the presence or absence of the auxin analogue NAA (100 nm). Scale, 50 µm. C) The proportion of side branches that give rise to ‘buds’ in wild type and *nog4-R* (no. buds formed per 15 cells), in the presence or absence of 100 nm NAA or 100 nm BAP (a cytokinin analogue). D) Comparison of the *PpCLF* transcript sequence in wild type and in the *nog4-R* mutant. Numbers indicate the relative position of the codon in the *PpCLF* transcript sequence (note the presence of a premature stop codon in *nog4-R* at position 104).

## Description

The colonization of land by plants was a pivotal moment in the history of life on Earth. The transition from aquatic to terrestrial environments was facilitated by the evolution of 3-dimensional (3D) growth; more specifically, the ability to rotate the division plane of an apical cell. Consequently, plants developed complex and diverse morphologies that enabled them to survive more effectively in harsh environments, located away from the water’s edge (Graham et al., 2000; Delwiche and Cooper 2015; Whitewoods et al., 2018) .

We recently developed the bryophyte *Physcomitrium patens* as a model system to genetically dissect 3D growth (reviewed elsewhere - Moody, 2019; Moody, 2022). *P. patens* has a protracted 2-dimensional (2D) filamentous growth phase (the tip growing protonema) that precedes the transition to 3D growth, and this can be continually maintained in tissue culture. The protonema initially comprises chloronemal cells, which emerge from spores, but later differentiates into caulonemal cells. From the latter emerge side branch initials that can form either secondary protonema (95%) or shoots (gametophores) that develop from gametophore initial cells (5%). To establish 3D growth, a gametophore initial cell divides obliquely to form an apical and a basal cell, which then both divide obliquely (perpendicularly to the first division plane) to form a 4-cell bud. The apical cell then undergoes two successive rotating divisions to specify an apical cell with a tetrahedral shape, which can divide to self-renew and generate the cells that comprise the leaf-like phyllids that wrap around the central axis of the gametophore in a spiral phyllotaxy (Harrison et al., 2009; Tang et al., 2020) ( Figure 1A ). A fine balancing act between auxin and cytokinin ensures that the correct number of gametophores are formed, cell walls are correctly oriented in developing gametophores, and that stemness of the tetrahedral apical cell is maintained (Ashton et al., 1979; Schulz et al., 2000; Thelander et al., 2019; Hyoung et al., 2020) . Important regulators of the initiation, establishment, and maintenance of 3D growth include PpAPB1-4 (Aoyama et al., 2012) , PpMACRO2 (Wang et al., 2020) , the glycerol-3-phosphate acyltransferases PpGPAT2 and PpGPAT4 (Lee et al., 2020) , Defective Kernel 1 (Perroud et al., 2014; Demko et al., 2014; Johansen et al., 2016; Perroud et al., 2020) , CLAVATA (Whitewoods et al., 2018; Cammarata et al., 2022) , the exocyst subunit PpSEC6 (Brejšková et al., 2021), the microtubule-associated protein targeting factor for Xklp2 (TPX2; Kozgunova et al., 2022), and NO GAMETOPHORES 1 (Moody et al., 2018) and NO GAMETOPHORES 2 (also known as PpHCT; Moody et al., 2021; Kreigshauser et al., 2021).

To identify additional regulators of 3D growth, we UV-mutagenized a wild-type strain of *P. patens* (Gd::GFP; Perroud et al., 2011) and screened for mutants that failed to establish 3D growth. As a result of this screen, we identified the ‘ *no gametophores 4 - Reference’* ( *nog4-R* ) mutant, which exhibits an array of striking developmental defects. Notably, in *nog4-R* mutants, side branch initials that normally give rise to filaments or buds are largely displaced by sporophyte-like indeterminate structures that can neither establish 3D growth nor develop gametophores ( Figure 1A ). We have observed three distinct developmental trajectories adopted by these sporophyte-like structures in the *nog4-R* mutant, referred to as the *lanceolate* , *unicorn* and *minotaur* . The *lanceolate* structure is derived from a small isodiametric gametophore initial cell that divides obliquely as in wild type, but then fails to establish a clearly defined apical and basal cell. Successive divisions predominantly occur perpendicularly to the first two division planes and is accompanied by a marked reduction in cell expansion. This results in the formation of a compact and bulbous structure that resembles the head of a lance, and in some cases, multiple lanceolate structures can form from a single gametophore initial cell. A *unicorn* is derived from a bulbous gametophore initial cell, but the first division is roughly parallel to the parent caulonemal cell and is not characteristically oblique. Subsequent divisions establish an apical cell that divides to self-renew but then yields cells from only two faces, seemingly in two continuous cell files (2D growth). A *minotaur* is derived from a gametophore initial cell that appears, at first, to follow the trajectory towards 3D growth; the first division is characteristically oblique, and the second division is perpendicular to the first division. However, the cell that would ordinarily transition into a rhizoid initial cell eventually switches fate to become an extra apical cell. Consequently, a bifurcation event is triggered, and this results in the formation of a two-horned bud with bilateral symmetry; 3D growth is never fully specified or established. We have observed that, new cell walls that form at the earliest stages of the development of either *lanceolate* , *unicorn* or *minotaur* structures are curved and wavy, like those observed in the *Δdek1* mutant (Perroud et al., 2014) . Furthermore, both *unicorn* and *minotaur* structures follow an indeterminate growth programme, and it is not unusual to detect structures that exceed 700 µm in length ( Figure 1A ) *.*

The *nog4-R* mutant phenotypes observed were highly reminiscent of those mutants with underlying defects in auxin and/or cytokinin signaling. Thus, we explored whether the *nog4-R* mutant could respond to auxin or cytokinin in an appropriate manner. We discovered that exogenous auxin treatment can strongly suppress the outgrowth of the indeterminate sporophyte-like structures to restore filamentous growth to the mutants ( Figure 1B ). This implies that the *nog4-R* mutant is defective in maintaining auxin homeostasis similarly to the recently described *nog2-R* mutant, which fails to inhibit supernumerary gametophore initial cell formation (Moody et al., 2021) . We went on to quantify the effects of auxin and cytokinin treatment on the proportion of side branches that are fated to become a bud; it has previously been shown that approximately one in 15 side branch initials are fated to become a gametophore in wild type (Aoyama et al., 2012; Perroud et al., 2014) . We performed an assay to calculate the proportion of side branches that initiate buds versus those that form filaments and compared wild type to the *nog4-R mutant* in the presence and absence of auxin or cytokinin. In wild type, we observed that the proportion of side branches that form buds is mildly reduced in response to auxin treatment but is strongly induced by cytokinin (approximately 60% of the filaments formed at least one bud and some formed as many as four buds). Conversely, in *nog4-R* , the proportion of side branches that form buds is extremely high in the absence of either auxin or cytokinin (approximately 95% filaments formed at least one bud and some formed as many as six buds), is strongly reduced in response to auxin treatment (approximately 60% filaments formed at least one bud and some formed as many as three buds) and is somewhat unaffected by cytokinin treatment ( Figure 1C ). These data suggest that endogenous levels of cytokinin within the *nog4-R* mutants may be disproportionately high, and that auxin treatment is required to readdress the balance between auxin and cytokinin.

It is known that the polycomb repressive complex 2 (PRC2) components FERTILIZATION INDEPENDENT ENDOSPERM (PpFIE) and CURLY LEAF (PpCLF) are required to suppress the formation of sporophyte-like apical cells within the gametophyte generation of *P. patens* (Mosquna et al., 2009; Okano et al., 2009; Pereman et al., 2016) . PpFIE and PpCLF are represented by single copy genes in *P. patens* , and disruption of either of these genes results in the formation of sporophyte-like apical cells in the gametophyte generation. In the gametophyte, these apical cells exhibit uncontrolled indeterminate growth. This is quite unlike a true sporophyte apical cell, which undergoes a brief determinate growth programme before a seta meristem is formed and subsequently a mature sporangium (Sakakibara et al., 2008) . We realised that the *nog4-R* mutant bore a striking resemblance to those mutants lacking a functional copy of either the *PpCLF* or *PpFIE* genes. Thus, we took a candidate gene approach to determine whether *nog4-R* contained UV-induced mutations within the coding sequences of either *PpCLF* or *PpFIE* . We discovered that the coding sequence of *PpFIE* aligned perfectly with the wild-type sequence and therefore did not contain any UV-induced mutations. However, a G>A transition generated a nonsense mutation (W ^104^ Ter) in the coding sequence of the *PpCLF* gene ( Figure 1D ). Thus, we have demonstrated that a mutated *PpCLF* gene was responsible for the *nog4-R* mutant phenotype.

In summary, loss of *PpCLF* function leads to the excessive formation of gametophore initial cells, and cell division orientation defects prevent the specification of a tetrahedral-shaped apical cell that is required for 3D growth. Furthermore, supernumerary gametophore initial cell formation can be suppressed by exogenous auxin treatment. Thus, *PpCLF* is required to specify 3D growth in an auxin-dependent manner.

## Methods

***Growth and propagation of P. patens tissues***

*P. patens* tissues were propagated on either BCD medium (bud induction) or BCD medium supplemented with 1 mM ammonium tartrate (routine propagation, BCDAT medium). BCD medium contained 250mg/L MgSO _4_ .7H _2_ O, 250mg/L KH _2_ PO _4_ (pH6.5), 1010mg/L KNO _3_ , 12.5mg/L FeSO _4_ .7H _2_ O, 0.001% Trace Element Solution (TES – 0.614mg/L H _3_ BO _3_ , 0.055mg/L AlK(SO _4_ ) _2_ .12H _2_ O, 0.055mg/L CuSO _4_ .5H _2_ O, 0.028mg/L KBr, 0.028mg/L LiCl, 0.389mg/L MnCl _2_ .4H _2_ O, 0.055mg/L CoCl _2_ .6H _2_ O, 0.055mg/L ZnSO _4_ .7H _2_ O, 0.028mg/L KI and 0.028mg/L SnCl _2_ .2H _2_ O) and 0.8 % agar, supplemented with 1 mM CaCl _2_ . For routine propagation, tissues were homogenized in water using an IKA T-25 basic Ultra Turrax® homogenizer, plated onto cellophane (A.A. Packaging Ltd.) overlaid BCDAT medium, and the grown at 24 ^o^ C with a 16 h: 8 h, light (300mmol m ^-2^ s ^-1^ ): dark cycle.

***UV mutagenesis***

A 1% Driselase solution (Sigma-Aldrich, Cat. No. D9515) was prepared in 8% mannitol and incubated at room temperature for 15 min before centrifugation at 3,300 xg for 3 min. The supernatant was filter sterilized through a 0.22 µm syringe filter into a sterile tube. To isolate protoplasts, the Gd::GFP marker line (Perroud et al., 2011) , grown for one week on cellophane overlaid BCDAT, was added to the Driselase solution, wrapped in foil and then gently agitated for 30-40 min at room temperature. The protoplast suspension was passed through a 100 µm cell strainer, pelleted by centrifugation at 120 xg for 3 min, and then washed twice with 8% mannitol (repeating the centrifugation in between each wash step). The cells were counted using a haemocytometer, and the cell density adjusted to 5x10 ^4^ cells mL ^-1^ . 1 mL of the cell suspension was then added to individual petri dishes containing cellophane overlaid PRMB medium (BCDAT supplemented with 10 mM CaCl _2_ , 6% mannitol and 0.5% glucose). These plates were then exposed to a 75000 µJ dose of UV radiation using a Stratalinker (lids off). Irradiated plates were incubated at 24 ^o^ C in the dark for 24-48 h and then transferred to the light for a further 2-3 weeks to allow protoplast regeneration. Individual regenerating protoplasts were transferred to wells of a 24 well plate containing BCD and grown under standard conditions for 4-6 weeks, and then phenotypically characterized. Those mutants that were unable to produce gametophores were selected for long term growth on BCD medium.

***Microscopy***

To check cell wall division orientation, tissues were grown for two weeks on BCD medium and then stained using 10 μgmL ^-1^ propidium iodide (PI) for 2 minutes. Tissues were then mounted in water and imaged using a Leica SP5 confocal microscope. PI was excited at 488 nm with 20% laser power, and a 63x water immersive lens was used in detecting PI at 600-630 nm. All other images were captured using either a Leica DMRB microscope or a Leica M165C stereomicroscope, equipped with a QImaging Micropublishing 5.0 RTV camera.

***Bud assays***

To quantify the proportion of side branches that give rise to filaments, tissues (wild type and *nog4-R* ) were homogenized and plated onto BCD plates supplemented with 100 nM 6-benzylaminopurine (BAP; synthetic cytokinin), 100 nM 1-naphthaleneacetic acid (NAA; synthetic auxin) or a minimal amount of 70% ethanol (solvent control). Three-week-old tissues were mounted on a slide in water to determine budding frequency. This was performed by determining the buds produced on side branches within a stretch of 15 caulonemal cells. Four biological replicates (colonies) were performed, and 10 filaments identified each time (n=40).

***Cloning of PpFIE and PpCLF***

RNA was extracted from one week old wild-type and *nog4-R* tissues grown on cellophane overlaid BCDAT, using the RNeasy Kit (Qiagen). RNA was DNase treated using TURBO DNase (Ambion) prior to cDNA synthesis using Superscript III Reverse Transcriptase (Invitrogen). Full length *PpFIE* and *PpCLF* transcripts were PCR amplified from cDNA using Verifi ^TM^ PCR Mix (PCRBIO) according to the manufacturer’s instructions. *PpFIE* was amplified using FIE_1.1K_FOR (ATGGCCCTGAAGCCTGC) and FIE_1.1K_REV (TCAAGACACAGCATCCCAGC), and *PpCLF* was amplified using CLF_3K_FOR (ATGGCGTCCTCCAGCTACG) and CLF_3K_REV (TTAAGCAACTTTCTGTGCTCGTC). PCR products were sequenced directly by Sanger sequencing (Source Bioscience).

## Reagents

**Table:** 

**STRAIN**	**GENOTYPE**	**AVAILABLE FROM**
Gd::GFP	*Physcomitrium patens*	Perroud et al., 2011 (PMID: 21366596)
*nog4-R*	*Physcomitrium patens*	Laura Moody (lead author) – line generated in this study
